# Nomogram for Predicting Bone Development State of Female Children and Adolescents–A Fast Screening Approach Based on Pubes Stages for Growth and Development

**DOI:** 10.3389/fped.2021.694958

**Published:** 2021-08-12

**Authors:** Ruoyu Yang, Liyan Wang, Chao Wu, Haihan Song, Jingyun Hu, Chen Jing, Qiaolin Zhang, Shihao Jia, Xunyi Lin, Yang Liu, Ming Cai, Xu Yan, Jian Wan, Hongbiao Wang

**Affiliations:** ^1^College of Rehabilitation Sciences, Shanghai University of Medicine and Health Sciences, Shanghai, China; ^2^Foundation of Shanghai Vocational College of Agriculture and Forestry, Shanghai, China; ^3^Central Lab, Shanghai Pudong New Area People's Hospital, Shanghai, China; ^4^Department of Talent Identification and Development, Shanghai Research Institute of Sports Science (Shanghai Anti-Doping Center), Shanghai, China; ^5^Faculty of Sports Science, Ningbo University, Ningbo, China; ^6^Institute for Health and Sport, Victoria University, Melbourne, VIC, Australia; ^7^Sarcopenia Research Program, Australian Institute for Musculoskeletal Science, Melbourne, VIC, Australia; ^8^Department of Emergency and Critical Care Medicine, Shanghai Pudong New Area People's Hospital, Shanghai, China; ^9^Department of Physical Education, Shanghai University of Medicine and Health Sciences, Shanghai, China

**Keywords:** bone development, nomogram, female, child and adolescent, pubes stages

## Abstract

**Objective:** To develop a nomogram for predicting bone development state (BDS) of female children and adolescents in a large scale.

**Methods:** Four hundred forty-seven female students were designated as the training cohort to develop the predictive model, whereas 196 female students were used as the validation cohort to verify the established model. Bone age, height, body mass, body fat percentage, and secondary sexual characteristics were recorded, and BDS was determined with the chronological age and bone age. Multivariate logistic regression was conducted to determine the factors, and nomogram was developed and validated with the training and validation cohorts, respectively.

**Results:** One hundred forty-seven female students were identified as BDS abnormal in the training cohort (32.9%), and 104 were determined in the validation cohort (53.1%). Age, height, weight, and pubes stage were selected for the predictive model. A nomogram was developed and showed a good estimation, with a C-index of 0.78 and a good calibration in the training cohort. Application of the nomogram to the validation cohort showed a similar C-index of 0.75 and a good calibration.

**Conclusion:** A nomogram for predicting bone development was developed, which can provide a relatively good estimation of BDS for female children and adolescents in Chinese metropolis.

## Introduction

Human growth and development is a rather complex process, affected by many factors. The level of growth and development can be reflected by some indicators in children and adolescents (1). Bone growth and development state is one of the factors, which can be determined by using the difference between bone age and chronological age.

Bone age, or skeletal age, needs to be assessed with specialized techniques, such as X-ray photography (2–4). However, application of X-ray photography in large-scale screening can be challenging in children and adolescents, due to the requirement of professionals and its cost associated with the interpretation of X-ray photography (5). Moreover, exposure to X-ray radiation might be harmful to children and adolescents. Therefore, alternative approaches are required to evaluate the bone development state (BDS) of children and adolescents in large-scale screening.

Some studies have reported that there was a high correlation between the secondary sexual characteristics and bone age or bone maturation state in children and adolescents (6–9). Testing of secondary sexual characteristics is relatively simple and feasible compared to X-ray photography; thus, it is worth exploring whether those characteristics can be used to predict bone age, so as to evaluate the BDS in children and adolescents.

In this study, we aimed to establish a model for predicting the BDS of female children and adolescents in Shanghai by using nomogram, which is commonly used in medical diagnosis as an approach for large-scale screening in Chinese metropolis.

## Materials and Methods

### Ethics Approval

This is part of a large study, which was approved by the ethics committee of School of Life Sciences Fudan University, Shanghai, China. Written consent forms were obtained from the parents of all participants.

### Participants

**Figure 1:**
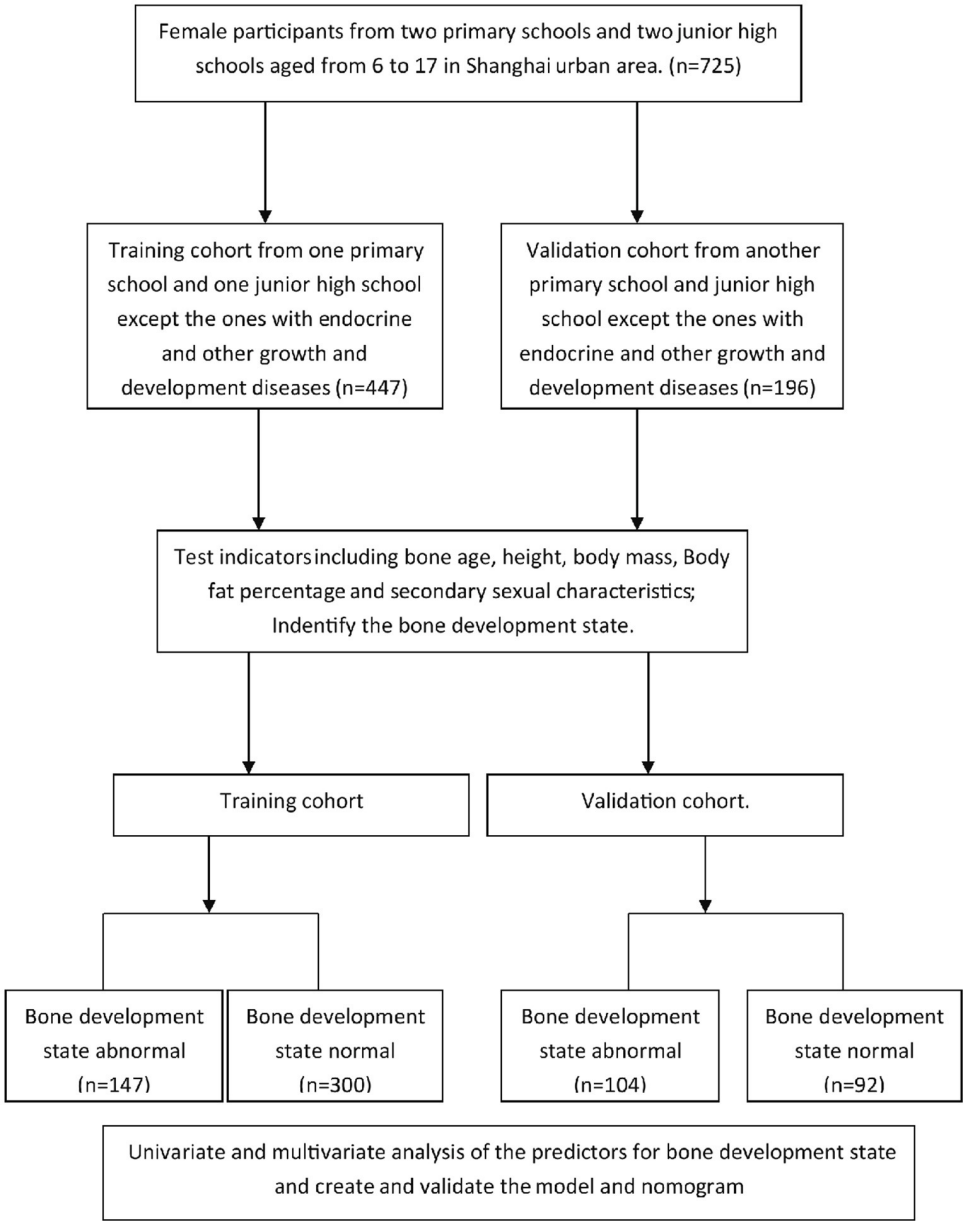
Flowchart of study design.

Seven hundred twenty-five female students (aged 6–17 years) from two primary schools and two junior high schools in the urban area of Shanghai were selected by cluster sampling. After exclusion of those with endocrine diseases or chronic diseases, 643 students were included in the study. Among them, 447 female students from one primary school and one junior high school were tested and studied to develop the predictive model as the training cohort, whereas 196 students from another primary school and another junior high school were used as the validation cohort to verify the model (Figure 1). The description of participants' characteristics is shown in Table 1.

**Table 1:** Participant characteristics.

**Variable**	**Cohort**, ***n*****(%)**		***p*-value**
	**Training (*n* = 447)**	**Validation (*n* = 196)**	
Age, y	11.1 (3.2)	11.0 (2.3)	0.60
Father's height, cm	173.3 (5.7)	173.0 (5.2)	0.34
Mother's height, cm	161.6 (5.3)	162.2 (5.8)	0.14
Height, cm	147.6 (14.8)	149.6 (12.6)	0.06
Body mass, kg	42.7 (14.1)	40.8 (11.5)	0.06
Body fat percentage, %	23.0 (7.0)	22.7 (5.8)	0.21
**Pubes**			
0	189 (42.3)	96 (49.0)	
1	15 (3.4)	10 (5.1)	0.06
2	12 (2.6)	6 (3.1)	
3	231 (51.7)	84 (42.8)	
**Breast**			
0	120 (26.8)	53 (27.0)	
1	40 (8.9)	22 (11.2)	
2	19 (4.3)	15 (7.7)	0.27
3	36 (8.1)	20 (10.2)	
4	232 (51.9)	86 (43.9)	

*Data are presented as mean (SD) for age, height, body mass, and body fat percentage. Data are presented in numbers (percentage) for pubes and breast*.

### Data Collection

Birth date and parents' height were obtained from each participant, as well as bone age, height, weight, body fat percentage, and secondary sexual characteristics (pubes and breast stage). Bone age was tested by a digital X-ray machine (CXDI-50G, Canon, Japan), and X-ray films were interpreted to determine the bone age with radius, ulna and short bone, (RUS); China (CHN) method (10–12). Height was measured with a height measuring instrument (Beijing Jianmin, China). Body weight and fat percentage were measured with a body composition instrument (Inbody 720, Inbody Corp., Korea). The secondary sexual characteristics were evaluated with Tanner stages method (13, 14). The BDS was determined by the difference between chronological age and bone age. The BDS was considered as normal if the difference was <2; otherwise, it was considered abnormal.

### Data Analysis

Data were analyzed using the SPSS 26.0 for Windows and the R Programming Language 4.0.5 version. Univariate logistic regression analysis was used to calculate the significance and strength of the association between each factor and BDS. Factor *p* < 0.10 was considered to have statistical significance and included in multivariate logistic regression analysis subsequently. Multivariate logistic regression analysis was also conducted to determine the factors independently associated with BDS. The results of multivariate analysis were reported as odds ratios and 95% confidence intervals, and *p* < 0.05 was considered to indicate statistical significance in the multivariate analysis. After these analyses, nomogram was built based on the results of the training cohort and validated with the validation cohort.

## Results

### Determination of BDS

Difference between chronological age and bone age was calculated, and the BDS was identified in both the training and the validation cohort. One hundred forty-seven participants (32.9%) from the training cohort and 104 participants (53.1%) from the validation cohort were identified as abnormal for BDS, respectively (Figure 1).

### Screening of Predictive Factors and Creating the Model

All variables listed in Table 1 were used for univariate logistic regression in the training cohort, and the ones with *p* < 0.10 were used for stepwise multivariate logistic regression analysis (Table 2). For the multivariate analysis, pubes stage was an independent factor (OR = 1.709, 95% CI = 1.166–2.504) associated with BDS in the training cohort. Finally, the model with pubes stage and three covariates including age, height, and body mass fitted the data well (Hosmer–Lemeshow test *p* = 0.578) (Table 3).

**Table 2:** Univariate logistic regression analysis of bone development state in the training cohort.

**Variable**	**Bone development normal (*n* = 300)**	**Bone development abnormal (*n* = 147)**	**OR (95% CI)**	***p*-value**
Age, y	10.2 (2.9)	12.9 (3.1)	1.338 (1.245–1.438)	<0.001
Father's height, cm	173.1 (5.7)	173.6 (5.6)	1.016 (0.981–1.053)	0.372
Mother's height, cm	161.0 (5.4)	160.9 (4.9)	0.994 (0.973–1.015)	0.551
Height, cm	144.5 (14.6)	154.0 (13.1)	1.051 (1.034–1.067)	<0.001
Body mass, kg	39.0 (12.7)	50.3 (13.6)	1.066 (1.048–1.084)	<0.001
Body fat percentage, %	21.3 (6.7)	26.4 (6.4)	1.129 (1.090–1.169)	<0.001
**Pubes**
0 (*n*)	162	27	1.873 (1.592–2.205)	<0.001
I (*n*)	13	2		
II (*n*)	12	0		
III (*n*)	113	118		
**Breast**
0 (*n*)	105	15	1.617 (1.405–1.862)	<0.001
I (*n*)	32	8		
II (*n*)	16	3		
III (*n*)	28	8		
IV (*n*)	119	113		

**Table 3:** Multivariate logistic regression analysis of bone development state in the training cohort.

**Variable**	**β**	**OR (95% CI)**	***p*-value**
Age	0.259	1.295 (1.116–1.503)	0.001
Height	−0.103	0.902 (0.861–0.944)	<0.001
Body mass	0.069	1.071 (1.035–1.109)	<0.001
Pubes	0.536	1.709 (1.166–2.504)	0.006

**Figure 2:**
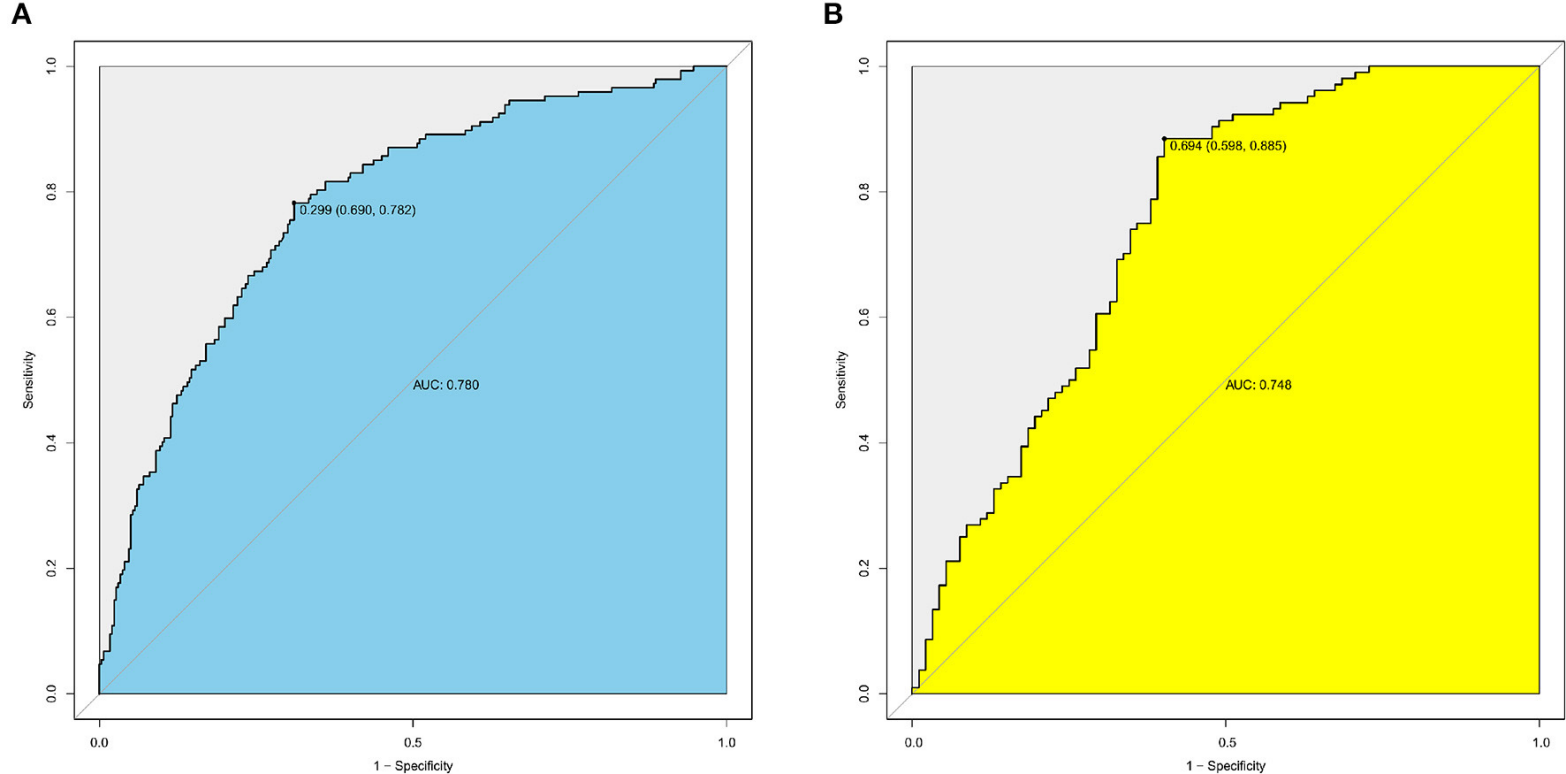
ROC analysis for the predictive model with data sets of training and validation cohorts. **(A)** The ROC curve for the predictive model with data set of training cohort, AUC = 0.78 (95% CI = 0.73–0.83). **(B)** The ROC curve for the predictive model with the data set of validation cohort, AUC = 0.75 (95% CI = 0.68–0.82).

The receiver operating characteristic (ROC) curve was analyzed and internally validated using bootstrap method for the predictive model, of which the area under the curve (AUC) was 0.78 (95% CI = 0.73–0.83). In the ROC curve, the threshold value was 0.299, with a specificity of 69.0% and sensitivity of 78.2% (Figure 2A). In the validation cohort, the predictive model showed an AUC value of 0.75 (95% CI, 0.68–0.82), with a threshold of 0.694 (specificity: 59.8%, sensitivity: 88.5%) (Figure 2B). The specificity, sensitivity, positive predictive value, negative predictive value, positive likelihood ratio, and negative likelihood ratio were calculated with the data of training cohort and validation cohort, respectively (Table 4).

**Table 4:** Accuracy of the model for predicting bone development state.

**Variable**	**Value (95% CI)**	
	**Training cohort**	**Validation cohort**
AUC	0.78 (0.73–0.83)	0.75 (0.68–0.82)
Specificity, %	69.0 (63.4–74.1)	59.8 (49.0–69.7)
Sensitivity, %	78.2 (70.5–84.4)	88.5 (80.3–93.6)
Positive predictive value, %	55.3 (48.3–62.1)	71.3 (62.6–78.8)
Negative predictive value, %	86.6 (81.5–90.5)	82.1 (70.4–90.0)
Positive likelihood ratio	2.5 (2.1–3.1)	2.2 (1.7–2.8)
Negative likelihood ratio	0.32 (0.23–0.43)	0.19 (0.11–0.33)

### Development and Validation of a BDS-Predicting Nomogram

**Figure 3:**
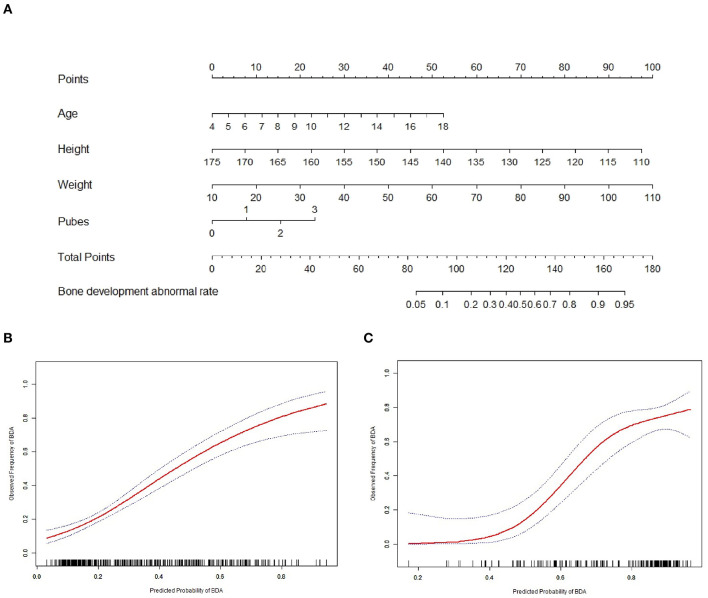
Nomogram for the predictive model and the calibration curves of the training and validation cohorts. **(A)** Nomogram for predicting bone development state of female children and adolescents. **(B)** The calibration curve of the nomogram for the training cohort. **(C)** The calibration curve of the nomogram for the validation cohort.

Based on the predictive model with pubes stage and the three covariates, a BDS-predicting nomogram was created (Figure 3A). The calibration curves of the training cohort and the validation cohort showed good agreement graphically, suggesting comparable BDS between the nomogram prediction and the bone age confirmed by the X-ray method (Figures 3B,C).

## Discussion

With the rapid development of social economy and the improvement of individuals' living quality, the growth and development characteristics of children and adolescents, especially those in big cities, have changed greatly compared to the past in China (15). The changes of environmental factors brought about by social development, such as air quality, water quality, and dietary habits, also have tremendous impacts on the growth and development of children and adolescents (16, 17). As a result, it is important to screen and evaluate the growth and development of children and adolescents regularly, so as to formulate appropriate health strategies and timely intervention for abnormal individuals. At present, in metropolitan area of China, such as Shanghai, the acquisition of growth and development status was screened and evaluated by using indicators such as height, body weight, and fat percentage (18). Bone age is also an important index for screening and evaluating growth and development, which can reflect BDS in children and adolescents. It has been applied in many fields, such as talent identification of juvenile athletes (19, 20). Bone age is a biological age, which is closely related to the growth and development of children and adolescents. In comparison with height, weight, and other indicators, which are mainly affected by chronological age but not the biological age, bone age may better reflect the growth and development state. Thus, it is reasonable to screen the growth and development level of children and adolescents. However, bone age is determined by X-ray photography; the associated risk of radiation hazards and difficulties in film interpretation make it not accessible for large-scale screening. With the development of digital photographing technology, the steps of photographing and imaging are greatly simplified, and individual X-ray photographing becomes more convenient. However, because of the complexity of X-ray film interpretation, the film interpreter should have certain professional skills and experience, and the film interpretation team should be relatively fixed, and the consistency should be checked frequently. If X-ray is used to screen a large population, the progress of film interpretation will be limited. Because of the rapid and convenient examination of secondary sexual characteristics, it is especially suitable for the screening of a large sample population. In the practical work of specific scenes, such as the primary screening of the growth and development of children and adolescents in the talent identification of athletes in Shanghai, the secondary sexual characteristics are generally used for the primary screening. In order to reduce the subjectivity of the second sexual characteristics examination, the staff are selected with the criteria of evaluating at least 10,000 cases every year. The work position is relatively fixed, and the consistency test between multiple people will be carried out regularly to ensure the accuracy of the examination.

Secondary sexual characteristics are highly correlated with bone age, and the examination is simple and feasible, with a unified international standard that has been implemented in China for several years (13, 14). Therefore, it is a better alternative to use the second sexual characteristics to predict bone age and BDS. The result of multivariate regression analysis in this study was also consistent with a previous study. Yang et al. (9) reported a correlation between bone age and secondary sexual characteristics in children and adolescents aged 7–18 years in Shanghai urban area. In that report, the bone age of male and female children and that of adolescents were significantly correlated with the secondary sexual characteristics, and the bone age of females had a high positive correlation with the pubes and breast stages with the same bone age criteria as our study. Regarding the correlation, the study established a univariate regression equation for bone age and pubes and breast of females, but did not take into account the influence of other confounding factors. In contrast, our current study was also based on the correlation between bone age and secondary sexual characteristics, but using the multiple regression analysis method and correcting for the confounding factors. Our study reflected the true relationship between bone age and secondary sexual characteristics of female children and adolescent more accurately. There were also similar reports in different ethnic populations. Susman et al. (21) reported the relationship between secondary sexual characteristics and the stage of growth and development in 9$\frac{1}{2}$- to 15$\frac{1}{2}$-year-old American white and black children and adolescents. In this study, each sexual maturity stage for genital (boys) or breast (girls) and pubes (boys and girls) was used to predict age, and the synchronous or asynchronous state of growth and development was determined (21). Compared with our study, Tanner original criteria were used in the identification and assessment of secondary sexual characteristics, so the results of the two studies could be compared horizontally. There was a correlation between secondary sexual characteristics and age (chronological age or bone age) in two different ethnic populations of female children and adolescents, and the synchronous or asynchronous state of growth and development was predicted, which were mostly consistent in two studies. The different results in the two studies lay on whether the dependent variable was chronological age or bone age. We believed that bone age could better reflect the real state of growth and development than chronological age. Growth and development are affected by race, social living environment, and economic development level, which may affect the reference data collected. With different reference data, the research result may not accurately reflect the real situation of the studied population or may be biased, which should be treated with caution. For example, the results obtained from the data of children and adolescents in less developed areas of China cannot be applied in metropolis such as Shanghai, because there are differences in social living environment and economic development level between the two populations.

Because the specific indicators of the secondary sexual characteristics are distinguished by gender, it is not appropriate to put the secondary sexual characteristics of both males and females into a predictive model at the same time. For this reason, this study focused only on females to establish a predictive model of BDS.

Nomograms are a pictorial representation of a complex mathematical formula widely used in biological, medical, and clinical fields especially in oncology (22–24). Generally, nomograms use biological and clinical variables, such as participants' age, height, weight, and other measurable variables, to establish a statistical prognostic or predictive model that can generate a probability of a clinical event (25). According to the standard method of establishing multivariate prediction model and nomogram (25, 26), a training and a validation cohort were used with to create the model and nomogram, which was verified by internal cross-validation and external data sets. Therefore, the predictive model has better reliability and repeatability in this study than that with only internal validation.

In this study, C-index (AUC) of nomogram was 0.78 in the training, and it was 0.75 in the validation cohort, which was in the range of 0.7–0.9, and had a relatively good discrimination ability. It has been reported that the AUC values of ROC curves in many cancer predictive models were from 0.6 to 0.9, which was consistent with the result of this study (25).

Based on our results, the model or nomogram has a good discrimination in the large-scale screening of the growth and development in urban female children and adolescents. Some possible limitations should be acknowledged for this study. Because bone development in children and adolescents will be affected by many aspects, there may be many confounding factors in the prediction model. The effect estimates in the predictive model are based on measured observation indices. Because of the limitations of some objective conditions, the measurement indicators may not be able to fully reflect all the bone development characteristics of female children and adolescents. There are no indicators such as blood routine examination or growth and development–related hormone levels that may be closely related to the bone development status. These indicators are likely to be confounding factors that affect the prediction of the model. If they are not corrected, the estimated accuracy of the model may be compromised. In the follow-up in-depth study, the research team will try to measure and acquire more indicators that may be associated with bone maturation state. Our study is a cross-sectional study of the population only in the urban area of Shanghai without multicenter study design. The research results may be applicable only to the urban area of Shanghai or China's metropolitan areas with similar development status. There may be regional and economic development restrictions on the applicability of the whole Chinese female children and adolescent population. In contrast, a similar study in the United States has done a better job in this regard. The multicenter population well-represented various regions and states all over the United States (21). Based on this limitation, we are planning to expand the sampling population to cities and villages with different economic development in different regions of China and strive to achieve the representative coverage and the universality of the application of the results.

In summary, we have developed a nomogram to predict BDS in female children and adolescents, based on pubes stage, chronological age, height, and body mass. The predictive model can provide a relatively good estimation of BDS for female children and adolescents in Chinese metropolis. It is worth exploring its application in other Chinese metropolises in and outside.

## Data Availability Statement

The raw data supporting the conclusions of this article will be made available by the authors, without undue reservation.

## Ethics Statement

The studies involving human participants were reviewed and approved by the ethics committee of School of Life Sciences Fudan University, Shanghai, China. Written informed consent to participate in this study was provided by the participants' legal guardian/next of kin.

## Author Contributions

RY, LW, and CW designed the study. RY, HS, JH, QZ, SJ, XL, and YL conducted the study and collected the data. RY, XY, and MC analyzed the data. RY and CJ drafted the manuscript. XY, JW, and HW revised the manuscript. All authors contributed to the article and approved the submitted version.

## Conflict of Interest

The authors declare that the research was conducted in the absence of any commercial or financial relationships that could be construed as a potential conflict of interest.

## Publisher's Note

All claims expressed in this article are solely those of the authors and do not necessarily represent those of their affiliated organizations, or those of the publisher, the editors and the reviewers. Any product that may be evaluated in this article, or claim that may be made by its manufacturer, is not guaranteed or endorsed by the publisher.
